# Effects of Ellagic Acid on Vaginal Innate Immune Mediators and HPV16 Infection In Vitro

**DOI:** 10.3390/molecules29153630

**Published:** 2024-07-31

**Authors:** Aornrutai Promsong, Jureeporn Chuerduangphui, Claire N. Levy, Florian Hladik, Surada Satthakarn, Wipawee Nittayananta

**Affiliations:** 1 Faculty of Medicine, Princess of Naradhiwas University, Narathiwat 96000, Thailand; aornrutai.p@pnu.ac.th; 2 Department of Microbiology, Faculty of Science, Kasetsart University, Bangkok 10900, Thailand; jureeporn.chu@ku.th; 3 Department of Obstetrics and Gynecology, University of Washington, Seattle, WA 98195, USA; clairel@uw.edu (C.N.L.); florian@uw.edu (F.H.); 4 Vaccine and Infectious Disease Division, Fred Hutchinson Cancer Research Center, Seattle, WA 98109, USA; 5 Faculty of Allied Health Sciences, Burapha University, Chonburi 20131, Thailand; surada.sa@buu.ac.th; 6 Faculty of Dentistry, Thammasat University, Pathum Thani 12120, Thailand

**Keywords:** ellagic acid, cytokines, hBD2, HPV-16, innate immune factors, phenolic phytochemical, SLPI

## Abstract

Ellagic acid (EA) is a phenolic phytochemical found in many plants and their fruits. Vaginal epithelial cells are the first line of defense against pathogen invasion in the female reproductive tract and express antimicrobial peptides, including hBD2 and SLPI. This study investigated the in vitro effects of EA (1) on vaginal innate immunity using human vaginal epithelial cells, and (2) on HPV16 pseudovirus infection. Vaginal cells were cultured in the presence or absence of EA, and the expression of hBD2 and SLPI was determined at both transcriptional and translational levels. In addition, secretion of various cytokines and chemokines was measured. Cytotoxicity of EA was determined by CellTiter-blue and MTT assays. To investigate the ability of EA to inhibit HPV16 infection, EA was used to treat HEK-293FT cells in pre-attachment and adsorption steps. We found significant increases in both *hBD2* mRNA (mean 2.9-fold at 12.5 µM EA, *p* < 0.001) and protein (mean 7.1-fold at 12.5 µM EA, *p* = 0.002) in response to EA. *SLPI* mRNA also increased significantly (mean 1.4-fold at 25 µM EA, *p* = 0.01), but SLPI protein did not. Secretion of IL-2 but not of other cytokines/chemokines was induced by EA in a dose-dependent manner. EA was not cytotoxic. At the pre-attachment step, EA at CC_20_ and CC_50_ showed a slight trend towards inhibiting HPV16 pseudovirus, but this was not significant. In summary, vaginal epithelial cells can respond to EA by producing innate immune factors, and at tested concentrations, EA is not cytotoxic. Thus, plant-derived EA could be useful as an immunomodulatory agent to improve vaginal health.

## 1. Introduction

Cervical cancer is the fourth most common type of cancer affecting women worldwide [1]. Human Papillomavirus (HPV) is the main etiological factor for cervical cancer. This virus is associated with the development of diseases ranging from benign warts to invasive cancer [2]. HPV16 is most prevalent in cervical cancer globally, followed by HPV18. Although approximately 80% of women acquire HPV infection by the age of 50, less than 1% of persistent infections progress to invasive cervical cancer [3]. Thus, vaginal innate immunity may play an important role against HPV infection and in preventing cervical cancer development.

The human vaginal epithelium is a portal to the external environment and a site for invasion by pathogens including bacteria, fungi, and viruses. Multiple layers of stratified squamous epithelial cells in the vaginal epithelium provide a physical barrier against microbial transmission. The epithelial cells secrete innate immune mediators including antimicrobial peptides, cytokines, and chemokines, which contribute to the mucosal defense against incoming pathogens [4,5]. The antimicrobial peptides inhibit both Gram-positive and Gram-negative bacteria such as *Staphylococcus aureus* and *Neisseria gonorrhoeae*, fungi (*Candida albicans*) and viruses (HIV-1) in part by the disruption of cell membranes and metabolic processes of the microbes. Human beta defensin 2 (hBD2) and secretory leukocyte protease inhibitor (SLPI) are powerful antimicrobial peptides [6,7]. hBD2 directly inhibits bacteria via membrane pore formation and hBD2 can suppress HIV-1 replication through G-protein coupled receptor-mediated signaling. Moreover, hBD2 can be induced in the presence of HPV infection [8,9]. The inhibitory effect of hBD2 on HPV has not been reported. SLPI plays multiple roles, including wound healing, antimicrobial activity by inhibiting proteases, and regulating inflammatory responses by suppressing nuclear factor-kappa B (NF-κB)-mediated inflammatory gene transcription downstream of Toll-like receptor (TLR) signaling. SLPI displays anti-HIV-1 activity as well as antifungal activity against *Candida albicans* and *Aspergillus fumigatus* [6,10]. Moreover, SLPI protects against HPV-induced squamous cancer by binding to annexin A2 [11,12]. In addition to antimicrobial peptides, chemokines such as CXCL8 play a crucial role in regulating immune function in the female reproductive tract. For example, it is important for attracting immune cells to the epithelium when homoeostasis is disrupted [13].

Plant-derived ellagic acid has a wide range of biological activities including anti-inflammatory, anti-oxidant, anti-cancer, and anti-bacterial action against *Helicobacter pylori* [14,15]. Additionally, ellagic acid is metabolized by the gut microbiota to urolithins, which protect against cancer and inflammatory diseases of the digestive tract [16]. Our previous study reported that ellagic acid can suppress replication of both R5- and X4-tropic HIV-1 viruses [17,18]. Interestingly, ellagic acid has the potential to stimulate the secretion of hBD2, SLPI, RANTES, and IL-2 in human gingival epithelial cells, whereas it downregulates the expression of CCL20, CXCL8, and CXCL5 [19]. A recent study reported that ellagic acid has the potential to prevent cervical cancer and enhance the clearance of HPV [20,21].

Based on these prior findings, we hypothesized that ellagic acid will also stimulate innate immune factor production in the human vagina, and that it might have anti-HPV effects. To investigate this, we measured (1) the effects of ellagic acid on the expression of innate immune mediators produced by vaginal epithelial cells, and (2) the effects of ellagic acid on the HPV16 pseudovirus infection in pre-attachment and adsorption steps. Additionally, we determined the cytotoxicity of ellagic acid on vaginal epithelial cells. The findings may serve as basic knowledge to support the development of a topical agent against HIV-1 infection and/or other pathogens invading the genital mucosa. 

## 2. Results

### 2.1. Cytotoxicity of Ellagic Acid in Primary Human Vaginal Epithelial Cells (HVEs)

**Figure 1:**
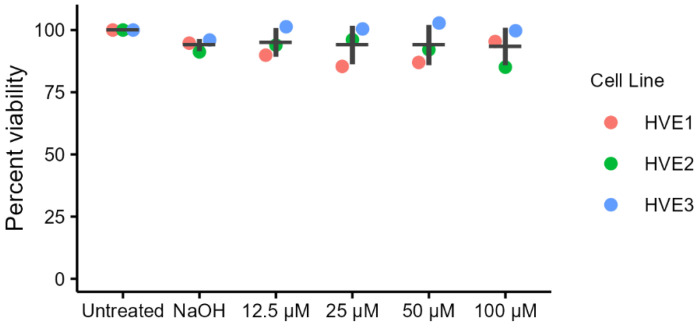
Mean ± S.D. of HVEs percent viability after 18 h of ellagic acid treatment measured by CellTiter-blue cell viability assay. Cell viability was calculated relative to untreated controls from the average of three technical replicates from three HVE donors. Averages from each donor are shown in red, green, and blue.

Cells were treated with four concentrations of ellagic acid in triplicate in three independent experiments, and cytotoxicity was assessed by CellTiter-Blue assay. No cytotoxicity was observed in HVEs after treatment with any of the ellagic acid concentrations; all conditions showed greater than or equal to 85% cell viability relative to untreated controls (Figure 1). 

### 2.2. Effects of Ellagic Acid on the Expression of Vaginal Innate Immunity

**Figure 2:**
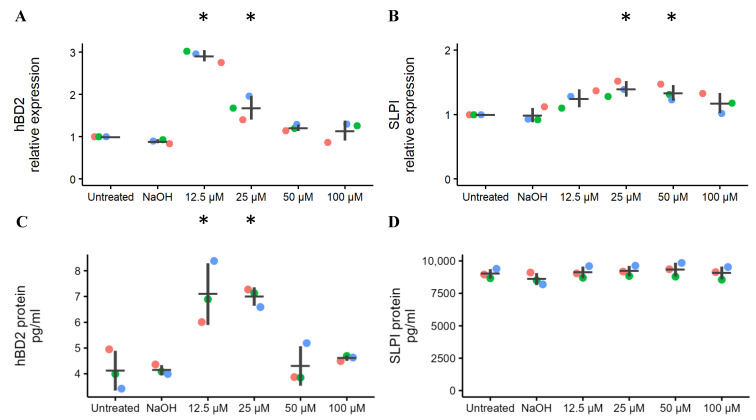
Mean ± S.D. of *hBD2* and *SLPI* mRNA and protein expression in HVEs. Ellagic acid was used at concentrations ranging from 12.5 to 100 µM to treat cells for 18 h. Cultures were grown in technical duplicates and the experiment was performed three times with HVEs from three donors, colored as in Figure 1. Averages from each donor are shown. (**A**,**B**) Expression of *hBD2* mRNA was significantly increased relative to the untreated control at 12.5 µM (*p* < 0.001) and 25 µM (*p* = 0.003), and *SLPI* mRNA was significantly increased at 25 µM (*p* = 0.01) and 50 µM (*p* = 0.04). Values were normalized to *GAPDH* mRNA expression. (**C**) Concentration of hBD2 protein was significantly increased at 12.5 µM (*p* = 0.002) and 25 µM ellagic acid (*p* = 0.003). (**D**) Concentration of SLPI protein was not significantly altered at any concentration of ellagic acid. Untreated cells served as negative controls. Cells stimulated with TNF-α served as positive controls and averaged 38,933.3 for hBD2 relative mRNA expression, 2.73 for SLPI relative mRNA expression, 22.4 pg/mL for hBD2 protein, and 9309.2 pg/mL for SLPI protein. * indicates *p* < 0.05 in when compared to untreated cells.

hBD2 and SLPI were measured at both transcriptional and translational levels. The experiments were performed in duplicate in three separate experiments. Compared to untreated cells, the expression of *hBD2* mRNA was increased on average 2.9-fold in response to ellagic acid at 12.5 µM (*p* < 0.001) and 1.7-fold at 25 µM (*p* = 0.003) (Figure 2A). The expression of *SLPI* mRNA increased on average 1.4-fold in response to 25 µM (*p* = 0.01) and 1.3-fold (*p* = 0.04) in response to 50 µM of ellagic acid (Figure 2B). hBD2 protein concentration in the culture media was significantly higher than the untreated condition in response to ellagic acid at 12.5 (*p* = 0.002) and 25 µM (*p* = 0.003) In contrast, the level of SLPI protein was not significantly different from the untreated cells at any concentration of ellagic acid (Figure 2C,D). 

### 2.3. Effects of Ellagic Acid on Cytokine and Chemokine Secretion Levels 

**Figure 3:**
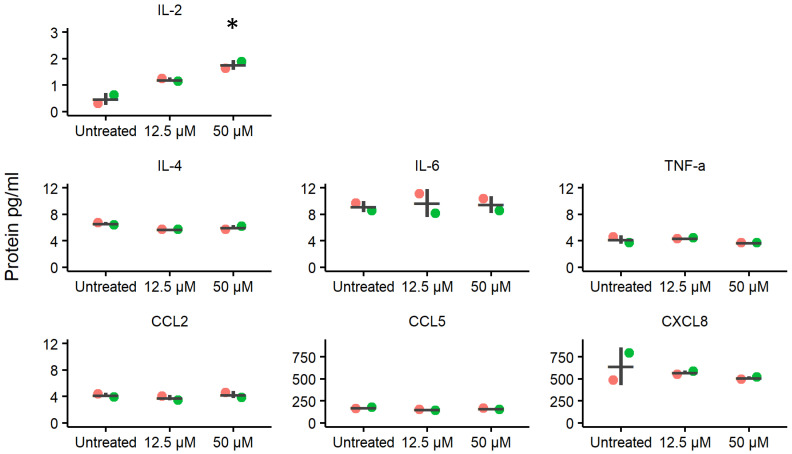
Mean ± S.D. of secreted immune mediator concentrations after treatment of HVEs from two donors (two biological replicates shown in red and green) with 0, 12.5, and 50 µM ellagic acid, as measured by Magnetic Luminex Performance Assay. IL-2 was significantly up-regulated (* = adjusted *p* = 0.014) upon exposure to 50 µM ellagic acid, relative to the untreated control. CXCL8 is also known as IL-8.

Cell culture supernatant was collected from HVE cultures in the presence or absence of ellagic acid. Seven out of ten immune mediators included in the assay were detected (IL-2, IL-4, IL-6, CXCL8, CCL2, CCL5, and TNF-α) as shown in Figure 3, whereas IL-1β, IFN-γ, and IL-10 were below detectable levels. The secretion of IL-2 increased in response to ellagic acid in a dose-dependent manner and the concentration of IL-2 in response to 50 µM of ellagic acid was significantly different from the untreated control condition after correcting for multiple comparisons (adjusted *p* = 0.014). No differential secretion of IL-4, IL-6, CXCL8, CCL2, CCL5, and TNF-α were apparent. The experiments were performed in duplicate in two separate experiments.

### 2.4. Effects of Ellagic Acid on HPV16 Pseudovirus Infection

**Figure 4:**
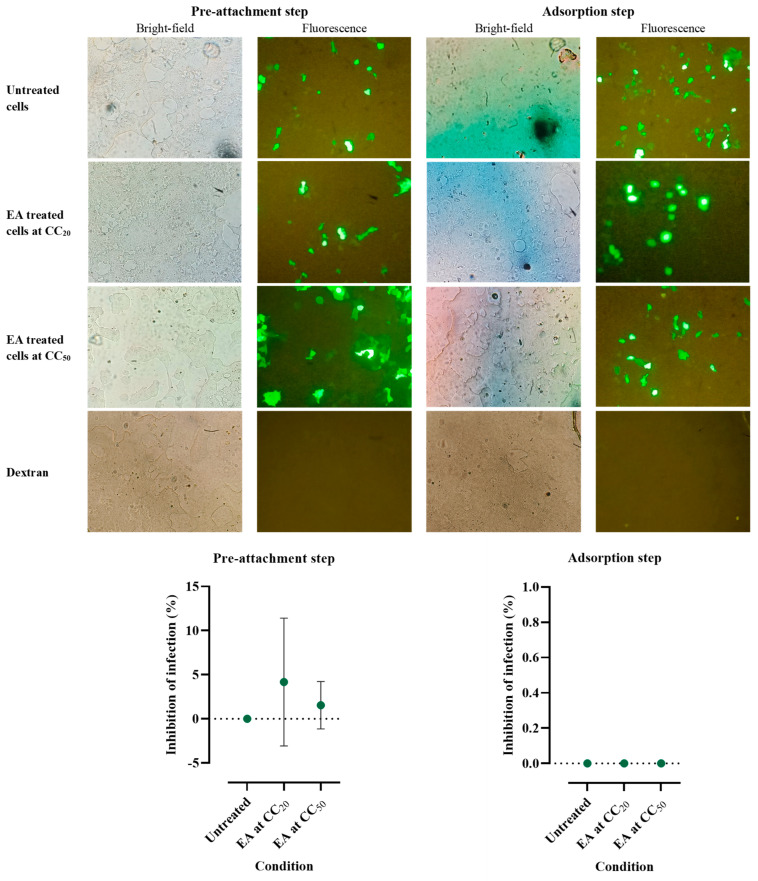
Fluorescence microscopic observation and mean ± S.D. of ellagic acid (EA) inhibitory effect on HPV16 pseudovirus infection in HEK-293FT cells. Effects of EA at CC_20_ and CC_50_ added either pre-HPV or post-HPV attachment were compared to untreated cells. The green-fluorescent cells represent HPV16 pseudovirus infection in untreated and treated cells. Dextran served as positive control. The cell images were captured using a microscope at 10× objective. The percentage of inhibition was calculated by the formula: 100 subtracting % infection.

In order to investigate the ability of ellagic acid to inhibit HPV16 infection, ellagic acid at 20% cytoxicity concentration (CC_20_) (0.56 ± 0.006 mM) and CC_50_ (4.28 ± 0.138 mM) were used to treat HEK-293FT cells in pre-attachment and adsorption steps. Experiments were performed in triplicate and the result is shown in Figure 4. Although this extract did not significantly inhibit HPV-16 infection, the pre-attachment step was found to show inhibitory effects with the following mean ± S.D. values: of CC_20_ (4.18 ± 7.24%) and CC_50_ (1.54 ± 2.68%).

## 3. Discussion

This study demonstrated the effects of ellagic acid on the expression of innate immune mediators produced by HVEs. Viability was at least 93% relative to untreated control upon exposure to concentrations of ellagic acid up to 100 µM, thus indicating no cytotoxic effects of ellagic acid on HVEs. Expression of both *hBD2* and *SLPI* mRNA was significantly up-regulated relative to untreated cells, with expression peaking in the middle of the range of concentrations we tested. This “bell-shaped” response curve could indicate a transition from agonistic to antagonistic effect as the dose of ellagic acid increases. This finding could be important when considering the clinical use of ellagic acid. hBD2 protein was also upregulated relative to untreated cells but ellagic acid did not affect the production of SLPI protein.

The secretion of hBD2 protein is induced by inflammatory substances and by various microorganisms, and it has potent antimicrobial properties against bacteria (*Escherichia coli*, *Staphylococcus aureus*), fungi (*Candida albicans*), and viruses [8,22]. hBD2 is inhibited by the hormone estradiol during the follicular phase of the menstrual cycle [23,24], which may render the female reproductive tract more vulnerable to infection. If the inflammatory effect observed was due to endotoxin or other contaminating substances in the cell culture medium, it would be expected to see a similar change in the untreated control condition. However, such an effect was not found. This may imply that the response is driven by exposure to ellagic acid. The finding that ellagic acid induces *hBD2* mRNA and protein secretion from genital epithelial cells may indicate an indirect protective activity against HIV-1 infection. One report indicated the ability of hBD2 to inhibit HIV-1 replication in macrophages at an early post-entry stage via G-protein coupled receptor-mediated signaling [9]. However, another report indicated that inhibition of HIV-1 by hBD2 in the mucosa may be limited to certain T cell subsets [25]. In our own prior reports, we showed that ellagic acid can directly inhibit both R5- and X4-tropic HIV-1 viruses in vitro [17,18], but in those studies we did not test for indirect antiviral effects via hBD2 induction. Thus, it remains to be determined whether ellagic acid can also enhance resistance to HIV-1 infection indirectly via induction of hBD2 in genital epithelial cells.

IL-2 secretion by HVEs increased in response to ellagic acid in a dose-dependent manner, but the secretion of other cytokines (IL-4, IL-6, CXCL8, CCL2, CCL5, and TNF-α) was not altered by the presence of ellagic acid. IL-2 stimulates proliferation of T lymphocytes and is involved in activation of lymphocytes and macrophages in preparation for attacking pathogens including *Candida albicans* and HIV-1 [26,27]. In healthy women, there are lower levels of IL-2 in the reproductive tract than in the oral mucosa [28]. HIV-1 infection is associated with dysregulation of cytokine production and can decrease the expression of protective IL-2 via interaction with HIV-1 gp160 [29,30] and HIV-1 Nef [31]. The increased secretion of IL-2 by HVEs in response to ellagic acid in the present study suggests that this compound may be able to counteract the suppression of IL-2 caused by HIV-1 infection [32]. On the other hand, protein expression of SLPI, IL-4, IL-6, CXCL8, CCL2, CCL5, and TNF-α by HVEs was not altered in the presence of ellagic acid, which suggests that it does not globally disturb the complex cytokine network of the mucosal immune system.

Of note, for HBD2 and SLPI we obtained both mRNA and protein measurements. In contrast to *hBD2*, the expression of *SLPI* mRNA did not correlate with its protein expression. According to other reports [33,34], the correlation between mRNA and protein expression levels can be as low as 40%, depending on the system and the regulation between transcription and translation. Furthermore, mRNA expression tends to be the more sensitive measurement for detecting changes and can happen much faster than changes in protein concentrations. Therefore, it is not unprecedented for mRNA and protein expression to differ.

According to previous reports, ellagic acid affects the expression of cytokines from several cell types, including peripheral blood mononuclear cells, pancreatic stellate cells, as well as liver and kidney cells [14,35]. However, the receptor pathways whereby ellagic acid is recognized by cells has never been studied. It was only reported that ellagic acid mediates its anti-inflammatory effects through the modulation of NF-κB activity by inhibiting IL-1β-induced nuclear translocation of p65 and p50 in human umbilical vein endothelial cells [36]. Perhaps some hints as to how ellagic acid works on innate immunity could be gleaned from studies with other phenolic compounds. A previous report found that polyphenols stimulate the immune system via multiple pathways [37]. Moreover, hydrolyzable tannins, including ellagic acid, were shown to reduce the secretion of innate inflammatory mediators such as TNF-α, IL-6, IL-1, PGE2, and MMPs, the main mechanism of which was explained via the impairment of NF-κB, COX-2 and MAPK pathways [38]. Additionally, phenolic acids similar to ellagic acid, such as curcumin and flavonoids, have been reported to down-regulate TLR-mediated signaling pathways [39,40]. We hypothesize that ellagic acid has a similar mechanism of action via inhibition of certain TLR-mediated pathways.

Lastly, we did not find a statistically significant inhibitory effect of ellagic acid on the HPV16 pseudovirus. Thus, while other studies indicated that ellagic acid has the potential to prevent cervical cancer and increase HPV clearance [20,21], we could not reproduce this anti-HPV effect in our in vitro model. Further experiments with additional HPV types, fully infectious virus, a wider range of infectious doses, and with other HPV-susceptible cell lines will be required to exclude an anti-HPV effect of ellagic acid. Because this would constitute the demonstration of the absence of an anti-HPV effect, parallel experiments with known HPV inhibitors will also be crucial [41]. 

The innate immunity-inducing activity of ellagic acid should be further evaluated in preclinical animal models where challenges with various pathogens can be performed in vivo and the therapeutic indices of various formulations can be tested. Ultimately, ellagic acid could transition to the clinic as a safe compound to restore vaginal immune homeostasis and/or as a preventative against some sexually transmitted infections. However, there still is a long path ahead towards reaching this goal. 

## 4. Materials and Methods

### 4.1. Ellagic Acid Preparation 

**Figure 5:**
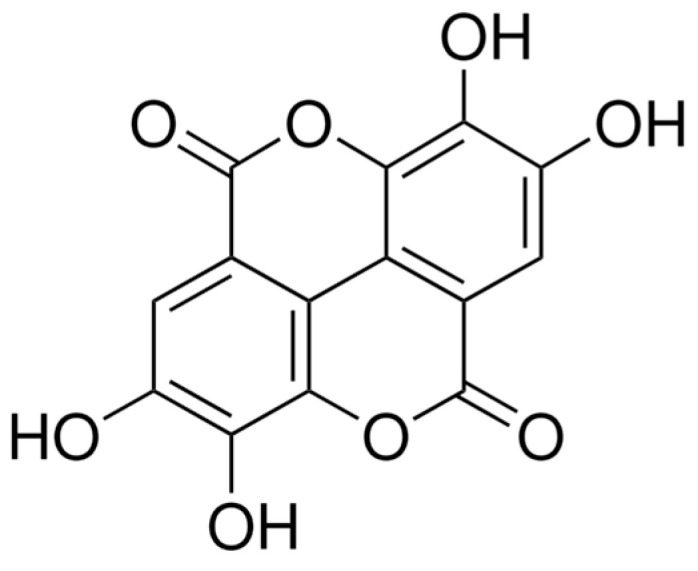
The structure of ellagic acid has a molecular formula of C_14_H_6_O_8_ and a molecular weight 302.19 g/mol (Sigma-Aldrich).

The ellagic acid used in this study was a powder derived from Chestnut bark, and its structure was shown in Figure 5 (Sigma-Aldrich, St. Louis, MO, USA). The powder was dissolved in 1 M NaOH and was diluted in cell culture media to various concentrations for experimental use. Diluted NaOH alone (0.3 mM) was used as vehicle control. 

### 4.2. Primary Human Vaginal Epithelial Cell Preparation

HVEs were co-cultured with 3T3-J2 mouse fibroblasts as feeders for HVE growth following protocols provided by the McBride laboratory (Laboratory of Viral Diseases at the National Institute of Allergy and Infectious Diseases, Bethesda, MD, USA), and methods originally described by Rheinwald and Green in 1975 [42] and refined by the McBride laboratory [43,44]. The fibroblast cells were a kind gift from Cary A. Moody, Department of Microbiology and Immunology, University of North Carolina-Chapel Hill, Chapel Hill, NC, USA.

### 4.3. Isolation of HVEs

Vaginal tissue for primary HVEs was obtained from healthy (i.e., non-cancerous) patients undergoing vaginal repair surgeries at the Department of Obstetrics and Gynecology, University of Washington Medical Center, Seattle, WA, USA. Although information on demographics and underlying disease is not available for these donors, all specimens came from surgeries performed for benign conditions such as uterine prolapse. There was no clinical suspicion of malignancy or the presence of a sexually transmitted disease. The tissue was collected from three participants, placed in ice-cooled calcium- and magnesium-free 1× PBS solution containing 100 U/mL penicillin, 100 µg/mL streptomycin and 2.5 µg/mL Amphotericin B (ThermoFisher Scientific, Waltham, MA, USA), and transported to the laboratory within 1 h of removal from the donor. Tissue harvesting and experimental procedures were approved by the Institutional Review Boards of the University of Washington and Fred Hutchinson Cancer Research Center. After the surgery, vaginal tissue was vigorously rinsed by swirling with 40 mL of sterile 1× DPBS in a 50 mL centrifuge tube. This procedure was repeated 10 times in 10 different tubes. Then the tissue was cut into approximately 8 mm diameter pieces in preparation for dispase digestion in 5 mL of a 25 U/mL dispase solution (BD Biosciences, Franklin Lakes, NJ, USA) and incubated at 4 °C for 16–18 h with gentle shaking. After incubation, epithelial sheets were peeled off the lamina propria using sterile forceps under a dissecting microscope, and lamina propria and subepithelium were discarded to exclude any contamination with stromal cells. The detached epithelial sheets were placed in a sterile 15 mL centrifuge tube containing 5 mL of cold 0.05% trypsin/EDTA (ThermoFisher Scientific) and incubated for 10–12 min in a 37 °C water bath with gentle shaking every few minutes to dissociate the cells from the sheets. Following trypsinization, 5 mL of warm DMEM/10% FBS was added to neutralize the trypsin/EDTA, and the solution was pipetted up and down for 5 min with a 10 mL serological pipette to obtain a single cell solution. Next, the cells were poured through a 100 µm cell strainer into a sterile 50 mL centrifuge tube and centrifuged at 4 °C for 5 min at 300× *g*. The supernatant was aspirated off and the pelleted cells were resuspended in sterile F-medium without epidermal growth factor (EGF). F-medium contains three volumes of Ham’s F-12 Nutrient mixture (ThermoFisher Scientific) and one volume of Dulbecco’s Modified Eagle Medium (DMEM) (high glucose) (ThermoFisher Scientific) supplemented with 5% fetal bovine serum (Gemini Bio-Products, West Sacramento, CA, USA), 0.4 µg/mL hydrocortisone (Sigma-Aldrich), 5 µg/mL insulin (GeminiBio-Products, West Sacramento, CA, USA), 8.4 ng/mL cholera toxin (Calbiochem, San Diego, CA, USA), 24 µg/mL adenine (Sigma-Aldrich), 100 U/mL penicillin, 100 µg/mL streptomycin, and 2 mM L-glutamine (ThermoFisher Scientific). This medium was sterilized with a 0.25 µm filter and stored at 4 °C. Prior to seeding the HVEs, T75 flasks were seeded at 13,000 cells/cm^2^ with irradiated (6000 rads) 3T3-J2 feeder cells. 3T3-J2 feeder cells were cultured in DMEM (ThermoFisher Scientific) with 10% fetal bovine serum, 2 mM Glutamine, 10 U/mL Penicillin, and 10 μg/mL Streptomycin.

The HVEs were plated at 3000 cells/cm^2^, cultured in 10 mL F-medium without EGF, and incubated in a humidified incubator at 37 °C and 5% CO_2_. The next day, the medium was changed to F-medium with 10 ng/mL EGF (ThermoFisher Scientific) and 10 µM of Rho kinase inhibitor Y-27632 dihydrochloride (Enzo Life Sciences, New York, NY, USA) and was replaced every two days. This culture medium was specifically formulated for the growth and adhesion of HVE; white blood cells will not survive in this medium, especially over more than one passage, so we can be confident that the cultures were purely HVE cells. In other studies, we also formally confirmed the identity of the HVE cells by the presence of cytokeratin staining and the absence of CD45 expression [45,46]. The HVE cells were used at passage 3, at about 70–80% confluence, for all experiments. Feeder cells were removed with 1× Versene (ThermoFisher Scientific) prior to subculture or experiments. In order to minimize donor-specific effects, all experiments used at least three HVE donors with technical duplicates.

### 4.4. Cytotoxicity of Ellagic Acid in HVEs

Cellular toxicity of ellagic acid in HVEs was determined by CellTiter-Blue cell viability assay (Promega, Madison, WI, USA). The assay measures metabolic activity as a proxy for cell viability and was performed according to manufacturer’s instructions. Briefly, the cells were seeded at a density of 1.2 × 10^4^ cells/well into 96-well plates in F-medium without EGF or Y-27632 and incubated for cell adhesion at 37 °C in a humid atmosphere of 5% CO_2_ overnight. After incubation, the medium was replaced with F-medium with 10 ng/mL EGF and 10 µM Y-27632 and incubated again overnight. On day three, the medium was replaced with 12.5 to 100 µM ellagic acid in F-medium or NaOH in F-medium alone as a vehicle control. A control blank (F-medium alone, no cells) and treated blank (each concentration of ellagic acid, no cells) were used to determine background fluorescence for the CellTiter-Blue assay. After 18 h of incubation at 37 °C, the CellTiter-Blue reagent was added directly to each well following the manufacturer’s instructions. The CellTiter-Blue fluorescence was excited at 560 nm and the emission at 590 nm was measured by SkanIt software 2.4.3 (RE) for Varioskan Flash. Relative cell viability was calculated as follows [47]: % Cell viability = $\left[\frac{ODtreated\;cells-ODtreated\;blank}{ODcontrol\;cells-ODcontrol\;blank}\right]\times 100$.

### 4.5. Treatment of HVEs with Ellagic Acid

We exposed HVEs to five concentrations of ellagic acid to test the hypothesis that ellagic acid can induce the expression of innate immunity in HVEs in vitro. 3T3-J2 irradiated fibroblast feeder cells were seeded at 5 × 10^4^ cells/well in 6-well plates and incubated for 24 h at 37 °C, 5% CO_2_ prior to irradiation. HVEs at passage three were plated at 5 × 10^4^ cells/well into the 6-well plates with the irradiated feeders in F-medium in the absence of EGF and Y-27632, and incubated for cell adhesion at 37 °C in a humidified atmosphere of 5% CO_2_ overnight. Next, the cell culture media was aspirated from the wells and the cells were treated with 0, 12.5, 25, 50, or 100 µM ellagic acid in F-medium (with EGF and Y27632), and incubated at 37 °C for 18 h. The negative control was HVEs with 0.3 mM NaOH in F-medium (vehicle control) and the positive control was HVEs stimulated with 100 ng/mL TNF-α (PeproTech 300-01A, PeproTech, Cranbury, NJ, USA). After incubation, the supernatant was collected and stored at −80 °C for secreted protein detection by enzyme-linked immunosorbent assay (ELISA) and Luminex technology. Feeder cells were removed with 1× Versene and the remaining HVEs were washed three times with 1× phosphate-buffered saline and lysed with buffer RLT (Qiagen, Redwood City, CA, USA). Total RNA was extracted using the RNeasy Mini Kit following the manufacturer’s protocol (Qiagen), including treatment with the RNase-Free DNase kit to remove genomic DNA contamination. Total RNA concentration was measured using the NanoDrop 1000 Spectrophotometer (ThermoFisher Scientific) and the RNA was stored at −80 °C prior to mRNA quantitation.

### 4.6. cDNA Preparation and Quantitative Real-Time PCR

After extraction, single stranded cDNA was synthesized from 500 ng of total RNA using the iScriptcDNA Synthesis Kit (Bio-Rad, Hercules, CA, USA) according to the manufacturer’s instructions. Controls without the reverse transcriptase enzyme were included in each experiment to confirm that the cDNA was free of genomic DNA contamination [19].

cDNA was analyzed for *SLPI* and *hBD2* targets (both FAM) by multiplex quantitative real-time PCR (qRT-PCR) using the TaqMan Gene Expression Master Mix (ThermoFisher Scientific). A target in glyceraldehyde 3-phosphate dehydrogenase (*GAPDH*) (VIC) was used as an endogenous control gene to determine the total amount of RNA (Table 1). The reactions were prepared in a total volume of 20 µL: 10 µL of TaqMan Gene Expression Master Mix (2×), 1 µL of 20× of target gene oligonucleotide primers and probes, 1 µL of 20× endogenous control gene oligonucleotide primers and probes (Integrated DNA Technologies, Coralville, IA, USA), 2 µL of cDNA and 6 µL of RNase-free water. No-template controls (H_2_O substituted for cDNA) were included. Amplification was performed using the 7900HT Fast Real-Time PCR system (Applied Biosystems, Waltham, MA, USA) under the following conditions: enzyme activation at 96 °C for 15 min followed by 40 cycles of denaturation at 95 °C for 15 s and annealing/elongation at 60 °C for 1 min. All amplifications were performed in duplicate, and average threshold cycle (C_t_) values were calculated. The levels of gene expression were normalized to *GAPDH* expression and calculated as relative expression compared to untreated control. ΔC_t_ was calculated as the difference in C_t_ between the target gene and the reference *GAPDH*. ΔΔC_t_ for each gene was calculated as the difference between the ΔC_t_ for treated cells and untreated cells and the fold change over untreated control was calculated as 2^−ΔΔCt^ [48].

### 4.7. Detection of Immune Mediator Concentrations in Culture Medium

Immune mediator concentrations in cell culture supernatant were measured by ELISA and Luminex technology. The cell culture supernatant was thawed on wet ice and centrifuged at 4 °C for 10 min at 221× *g* prior to the assays to pellet any solids remaining in the supernatant [19].

### 4.8. hBD2 and SLPI Immunoassays

hBD2 and SLPI proteins were quantified by ELISA (hBD2: Alpha Diagnostic, San Antonio, TX, USA, SLPI: Quantikine ELISA, R&D Systems, Minneapolis, MN, USA). The experiments were performed in duplicate following the manufacturer’s instructions. Absorbance of light at 450 nm was measured using Skanlt Software 2.4.3 (ThermoFisher Scientific). Standard curves of purified recombinant hBD2 and SLPI were used to calculate the protein concentration in the experimental samples [19].

### 4.9. Multiple Human Cytokines and Chemokines by Luminex Assay

The cytokines and chemokines were measured in multiplex by the Magnetic Luminex Performance Assay in the Human Cytokine Premixed Kit A (R&D Systems). The following analytes were quantified: IFN-γ, IL-1β, IL-2, IL-4, IL-6, IL-8, IL-10, CCL2, CCL5, and TNF-α. Luminex technology is based on the “antigen–antibody sandwich” principle. The sample is combined with magnetic beads that have been pre-coated with antibodies against the analytes of interest, followed by a set of biotinylated antibodies also against the target analytes, thus creating a “sandwich” with antibodies on either side of the analyte. For detection, the biotin on the antibody–antigen sandwich is bound by streptavidin conjugated to the phycoerithin (PE) fluorochrome. PE fluorescence is quantified by light-emitting diode. The Luminex assay was performed in duplicate according to the manufacturer’s instructions. The plates were read on a CS1000 Autoplex analyzer (PerkinElmer, Waltham, MA, USA) and Luminex xMAP^TM^ Technology (Luminex Corporation, Austin, TX, USA).

### 4.10. Cytotoxicity of Ellagic Acid in HEK-293FT Cells

Human embryonic kidney 293FT (HEK-293FT) cells purchased from Invitrogen were maintained in DMEM supplemented with 10% fetal bovine serum (Gibco, Grand Island, NY, USA) and 40 μg/mL gentamicin, 2.5 μg/mL fungizone, 100 unit/mL penicillin G, and 100 μg/mL streptomycin in a 5% CO_2_ incubator at 37 °C.

HEK-293FT cells was seeded at a density of 10,000 cells/well into a 96-well plate and incubated for 24 h in 5% CO_2_ incubator at 37 °C. Different concentrations of ellagic acid in completed medium were then added to the cells and continuously maintained in 5% CO_2_ incubator at 37 °C for 24 h. Ten microliters of 5 mg/mL MTT solution were loaded into the cells per well and incubated for at 37 °C for 4 h. Formazan was dissolved in DMSO after removing the culture medium. The absorbance was measured at 540 nm.

### 4.11. Production of HPV16 Pseudovirus

HEK-293FT cells at 60% confluence were transfected using Lipofectamine 2000 (Invitrogen) for 6 h with pfWB (green fluorescence-expressing vector) and p16SheLL (HPV16L1/L2-expression vector), which was kindly provided by John T. Schiller (Laboratory of Cellular Oncology, Bethesda, MD, USA). Transfected cells were harvested and lysed using 0.05% BriJ 58 and 0.01% RNase in PBS/MgCl_2_. After incubation at 37 °C for 24 h, the pseudovirus was measured for titer and stored at −80 °C until use. The method was modified from a previous study [49].

### 4.12. HPV Attachment Assays

#### 4.12.1. Ellagic Acid Added to HPV before HPV Attachment to Cells

HEK-293FT cells at a density of 4000 cells/well were seeded to a 96-well plate and maintained for 24 h. HPV16 pseudovirus at multiplicity of infection (MOI) 0.05 was pre-incubated with ellagic acid at CC_20_ or CC_50_ at 37 °C for 1 h. The mixture was added to the cells and maintained at 37 °C for 4 h. Untreated cells and cells treated with 400 µM dextran served as positive controls. The cells were then maintained in completed medium at 37 °C for 48 h after removing the unbound pseudovirus. The cells were collected and counted using a hemacytometer under a fluorescent microscope (Olympus BX51, Olympus Co., Ltd., Tokyo, Japan).

#### 4.12.2. Ellagic Acid Added to Cells after HPV Attachment to Cells

HEK-293FT cells at a density of 4000 cells/well were seeded to a 96-well plate and maintained for 24 h. The HPV16 pseudovirus was added to the cells at 20 °C for 2 h. After eliminating the unbound pseudovirus, ellagic acid at CC_20_ or CC_50_ was added to the cells and incubated at 37 °C for 48 h. Untreated cells and cells treated with 400 µM dextran served as positive controls. The cells were collected and observed under a fluorescent microscope (Olympus BX51, Olympus Co., Ltd., Tokyo, Japan).

### 4.13. Statistical Analysis

Results were recorded as mean ± S.D. of differences in gene or protein expression between treated cells and untreated cells in duplicate cultures from two to three separate experiments. The data were analyzed using One-Way ANOVA for the differences between groups at various concentrations. If the results of the ANOVA were statistically significant at α = 0.05, post hoc tests were performed for individual comparisons, again with a significance threshold of α = 0.05. Paired *t*-tests were performed for each cytokine to determine differences between untreated cells and cells exposed to 12.5 or 50 µM ellagic acid. The resulting *p*-values were adjusted for multiple comparisons by multiplying the *p*-value by the total number of t-tests performed. Calculations were performed using Microsoft Office Professional Plus 2013, GraphPad Prism 10.1.1 (GraphPad Software, La Jolla, CA, USA), and R 4.4.0 using RStudio [50,51]. Plots were made with RStudio 2024.04.1+748 [52,53,54,55,56,57].

## 5. Conclusions

Ellagic acid stimulated the gene and protein expression of hBD2 by human vaginal epithelial cells and induced their secretion of IL-2. Both hBD2 and IL-2 play important roles in mucosal innate immune responses. The mechanisms involved in the sole induction of IL-2 remain unknown. Future studies should be performed to investigate this as well as to explore any adverse effects of ellagic acid on the beneficial vaginal microbiome, specifically *Lactobacilli*. As of this research, we were not able to find any peer-reviewed studies of how ellagic acid may impact the vaginal microbiome. It should also be investigated if ellagic acid can be metabolized by the vaginal microbiota and protect against cancer and inflammatory diseases of the female genital tract, as has been reported for the gastrointestinal tract [14,15,16]. Taken together, our findings suggest that ellagic acid could be further tested and developed as an antimicrobial agent to prevent infections in the female genital tract. Ellagic acid is already used in traditional medicine; therefore, future research should consider the effect of ellagic acid in traditional preparations as well as its potential utility in evidence-based medicine.

## Figures and Tables

**Table 1:** Primer and probe sequences used for qRT-PCR.

**SLPI** **(NM_003064)** **Exon Location: 3–4**	Forward primer: 5′-CAAGCGTGACTTGAAGTGTTG-3′ Reverse primer: 5′-GAAAGGACCTGGACCACAC-3′ Probe: 5′-/56-FAM/AGGAGATAC/ZEN/AAGACCCTCATGGCTGA/3IABkFQ/-3′
**hBD2 (DEFB4A)** **(NM_004942)** **Exon Location: 1–2**	Forward primer: 5′-TCCTGGTGAAGCTCCCA-3′ Reverse primer: 5′-CGCCTATACCACCAAAAACAC-3′ Probe: 5′-/56-FAM/AGGAGATAC/ZEN/AAGACCCTCATGGCTGA/3IABkFQ/-3′

## Data Availability

Data are available from the corresponding author upon reasonable request.

## Abbreviations

hBD2: human beta defensin 2; SLPI: secretory leukocyte protease inhibitor; HVEs: human vaginal epithelial cells; IFN-γ: interferon gamma; IL-1β: interleukin 1 beta; IL-2: interleukin 2; IL-4: interleukin 4; IL-6: interleukin 6; CXCL8: C-X-C motif chemokine ligand 8, IL-8; IL-10: interleukin 10; CCL2: C-C motif chemokine ligand 2; CCL5: C-C motif chemokine ligand 5, RANTES (regulated upon activation, normal T-cell expressed and secreted); TNF-α: tumor necrosis factor alpha.
